# N-acetylcysteine Decreases Fibrosis and Increases Force-Generating Capacity of *mdx* Diaphragm

**DOI:** 10.3390/antiox8120581

**Published:** 2019-11-24

**Authors:** David P. Burns, Sarah E. Drummond, Dearbhla Bolger, Amélie Coiscaud, Kevin H. Murphy, Deirdre Edge, Ken D. O’Halloran

**Affiliations:** 1 Department of Physiology, School of Medicine, College of Medicine and Health, University College Cork, T12 XF62 Cork, Ireland; 2 Department of Physiology, School of Medicine, Trinity Biomedical Sciences Institute, Trinity College Dublin, The University of Dublin, D02 R590 Dublin, Ireland

**Keywords:** antioxidant, N-acetylcysteine, DMD, *mdx*, diaphragm, intercostal, interleukin-6, interleukin-1β, fibrosis

## Abstract

Respiratory muscle weakness occurs due to dystrophin deficiency in Duchenne muscular dystrophy (DMD). The *mdx* mouse model of DMD shows evidence of impaired respiratory muscle performance with attendant inflammation and oxidative stress. We examined the effects of N-acetylcysteine (NAC) supplementation on respiratory system performance in *mdx* mice. Eight-week-old male wild type (*n* = 10) and *mdx* (*n* = 20) mice were studied; a subset of *mdx* (*n* = 10) received 1% NAC in the drinking water for 14 days. We assessed breathing, diaphragm, and external intercostal electromyogram (EMG) activities and inspiratory pressure during ventilatory and non-ventilatory behaviours. Diaphragm muscle structure and function, cytokine concentrations, glutathione status, and mRNA expression were determined. Diaphragm force-generating capacity was impaired in *mdx* compared with wild type. Diaphragm muscle remodelling was observed in *mdx*, characterized by increased muscle fibrosis, immune cell infiltration, and central myonucleation. NAC supplementation rescued *mdx* diaphragm function. Collagen content and immune cell infiltration were decreased in *mdx* + NAC compared with *mdx* diaphragms. The cytokines IL-1β, IL-6 and KC/GRO were increased in *mdx* plasma and diaphragm compared with wild type; NAC decreased systemic IL-1β and KC/GRO concentrations in *mdx* mice. We reveal that NAC treatment improved *mdx* diaphragm force-generating capacity associated with beneficial anti-inflammatory and anti-fibrotic effects. These data support the potential use of NAC as an adjunctive therapy in human dystrophinopathies.

## 1. Introduction

Skeletal muscle weakness is a major feature of Duchenne muscular dystrophy (DMD) due to a deficiency in the protein dystrophin [1]. In healthy muscle, dystrophin has a structural role in supporting muscle fibres during repeated cycles of muscle contraction, acting to limit the mechanical stress applied to muscle fibres [2,3]. In the absence of dystrophin, such as in DMD, skeletal muscle fibres have increased fragility, resulting in fibre damage. Common pathological features of dystrophic muscle include myonecrosis, inflammation, and oxidative stress [4,5]. Due to profound muscle weakness and damage, children with DMD have difficulty with ambulation in their early years, necessitating assistive devices.

Respiratory system morbidity occurs in DMD due to weakness of the respiratory musculature [6], the end effectors of breathing. Respiratory function is compromised in boys with DMD, with reductions in forced vital capacity [7,8] and impaired inspiratory muscle strength [6,9], evident from a young age. Impaired respiratory function is most prominent during sleep, evidenced by an increased risk of sleep disordered breathing in children with DMD [10]. Many patients require ventilatory support during sleep to maintain blood gas homeostasis. Cough and airway clearance manoeuvres are compromised in DMD due to reduced respiratory muscle strength, such that patients are more susceptible to aspiration pneumonia [11]. Therapies aimed at improving respiratory muscle strength in DMD are necessary and attractive, in order to maintain adequate ventilation and prevent respiratory failure, a leading cause of mortality in DMD, notwithstanding recent advances in the respiratory support of DMD boys.

Studies of the respiratory system in the *mdx* mouse, a preclinical model of DMD, have documented profound diaphragm muscle weakness and structural remodelling from a young age as a consequence of dystrophin deficiency [12,13,14,15,16]. Inflammatory markers such as immune cell infiltration and cytokine concentrations are increased in *mdx* diaphragm, as well as the abundance of collagen deposits [17]. Moreover, indices of oxidative stress including lipid peroxidation and superoxide levels are elevated in *mdx* diaphragm compared with control muscle [18]. Inflammation and high levels of reactive oxygen species (ROS) can culminate in skeletal muscle damage leading to poor physiological performance [19]. Oxidative stress is a recognized feature of respiratory disorders including DMD. Targeting oxidative stress within muscle by reducing the bioavailability of ROS or boosting endogenous antioxidant stores are attractive adjunctive therapies, particularly in conditions where redox imbalance presents and contributes to muscle pathology [20,21]. We have previously demonstrated that administration of a superoxide scavenger (Tempol) to *mdx* mice for two weeks restores metabolic enzyme activities and improves diaphragm muscle force-generating capacity [22]. It has been shown by others that Tempol supplementation reduces myonecrosis and inflammation in the diaphragm and biceps brachii muscles of *mdx* mice [23]

N-acetylcysteine (NAC) is a dietary antioxidant and precursor to glutathione, an endogenous antioxidant, safe for use in humans. Interestingly, NAC is a mucolytic agent and is commonly used in patients with cystic fibrosis and chronic obstructive pulmonary disease. Previous studies from our group have demonstrated beneficial effects of NAC supplementation on respiratory muscle function in animal models of respiratory disease [24,25,26]. Studies utilising NAC as a potential therapeutic for dystrophic disease have yielded promising results. Pinniger et al. (2017) reported improved normalized grip strength and extensor digitorum longus (EDL) force in *mdx* mice supplemented with 2% NAC in the drinking water for 6 weeks [27]. In a separate study, intraperitoneal injections of NAC in 14 day old *mdx* mice for 14 days reduced tumour necrosis factor-α (TNF-α) and lipid peroxidation levels in *mdx* diaphragm [28]. Terrill et al. (2012) reported that NAC administered in the drinking water (1% NAC for 6 weeks or 4% NAC for one week) prevented exercise-induced myonecrosis in quadriceps muscle of *mdx* mice [29]. Studies by Whitehead et al. (2008) determined that 1% NAC in the drinking water for 6 weeks reduced the concentration of ROS and decreased damage in EDL muscle of *mdx* mice [30]. Collectively, these studies support the use of NAC to target muscle damage mediated by oxidative stress in *mdx* mice, but no studies to date have assessed the efficacy of NAC in ameliorating respiratory system deficits in *mdx* mice.

In the current study, we set out to perform a broad and thorough assessment of the effects of NAC supplementation on respiratory system performance in young (8-week-old), male *mdx* mice. Six-week-old *mdx* mice were treated with 1% NAC in the drinking water for 14 days. We hypothesized that NAC would have beneficial effects on dystrophic respiratory muscle, leading to preserved respiratory system performance.

## 2. Materials and Methods

### 2.1. Ethical Approval

Procedures on live animals were performed under licence in accordance with Irish and European directive 2010/63/EU following ethical approval by University College Cork (AEEC no. 2013/035). Experiments were carried out in accordance with guidelines laid down by University College Cork’s Animal Welfare Body, and conform to the principles and regulations described by [31].

### 2.2. Experimental Animals and N-acetylcysteine (NAC) Treatment

Male wild type (WT; C57BL/10ScSnJ; *n* = 10) and *mdx* (C57BL/10ScSn-Dmd^mdx^/J; *n* = 20) mice were purchased from the Jackson Laboratory (Bar Harbor, ME, USA) and were housed at University College Cork’s animal facility. Animals were housed conventionally in temperature- and humidity-controlled rooms, operating on a 12 h light:12 h dark cycle with food and water available *ad libitum*. Mice were assigned to three different experimental groups: wild type, *mdx* and *mdx* + NAC. The *mdx* + NAC group received 1% N-acetylcysteine (Sigma-Aldrich, Wicklow, Ireland) in the drinking water for 14 days, beginning at six weeks of age. Drinking water containing NAC was pH matched to control water (pH 8). Mice were studied at eight weeks of age. We performed a thorough assessment of respiratory performance, with measurements of breathing and ventilatory capacity in response to chemoactivation, recordings of thoracic inspiratory pressure and respiratory muscle electromyography (EMG) in vivo and respiratory muscle function tests ex vivo. Diaphragm muscle tissue was collected for structural analysis using histological and immunofluorescence techniques. Diaphragm glutathione status and mRNA expression were examined. Cytokine concentrations were determined in diaphragm and plasma samples.

### 2.3. Whole-Body Plethysmography

Whole-body plethysmography was used to assess respiratory flow in unrestrained, unanaesthetized mice. Mice were introduced into plethysmograph chambers (Model PLY4211; volume 600 mL, Buxco Research Systems, Wilmington, NC, USA) and were allowed an acclimation period typically lasting 40–60 min, with room air passing through each chamber (1 L min^−1^).

Experimental protocol: Following acclimation, during confirmed periods of quiet rest, a 20-min baseline recording was performed in normoxia. This was followed by central and peripheral chemoreceptor stimulation with hypercapnic-hypoxia (10% O_2_ & 6% CO_2_) for 5 min, to examine maximum chemoactivated breathing. Respiratory parameters including respiratory frequency (*f*_R_), tidal volume (*V*_T_) and minute ventilation (*V*_E_) were recorded on a breath-by-breath basis for analysis offline.

Data analysis: Baseline normoxic ventilation was determined as an average of the baseline period. For hypercapnic-hypoxia, ventilatory measurements were taken during the final (5th) minute of the challenge. *V*_T_ and *V*_E_, were normalized for body mass (g).

### 2.4. Diaphragm and External Intercostal EMG and Oesophageal Pressure Recordings

Mice were anaesthetized with 5% isoflurane in 60% O_2_ (balance N_2_) followed by urethane (1.7 g kg^−1^ i.p.). Wild type (*n* = 10), *mdx* (*n* = 10) and *mdx* + NAC (*n* = 10) mice were then placed in the supine position and were gradually weaned from the isoflurane. Body temperature was maintained at 37 °C via a rectal probe and thermostatically-controlled heating blanket (Harvard Apparatus, Holliston, MA, USA) for the duration of the experiment. Supplemental anaesthetic was administered as necessary to maintain a surgical plane of anaesthesia. Depth of anaesthesia was determined by assessment of pedal withdrawal reflex to noxious pinch. Peripheral capillary O_2_ saturation (SpO_2_) was monitored using a pulse oximeter clip (MouseOx^TM^, Starr Life Sciences Corporation, Oakmount, PA, USA). A thigh of each mouse was shaved and the pulse oximeter was attached. A tracheotomy was performed in the mid-cervical region. A bias flow of supplemental O_2_ (FiO_2_ = 0.60) was provided to all animals during baseline conditions. End-tidal carbon dioxide (ETCO_2_) was measured using a MicroCapStar (CWE, Ardmore, PA, USA). A pressure-tip catheter (Mikro-Tip, Millar Inc., Houston, TX, USA) was inserted into the mouth and positioned in the thoracic oesophagus for the assessment of oesophageal pressure, an index of intrapleural sub-atmospheric pressure generated by the respiratory musculature during inspiration [13]. Oesophageal recordings displayed phasic sub-atmospheric pressure swings during inspiration. For the measurement of diaphragm EMG, a concentric needle electrode (26G; Natus Manufacturing Ltd., Galway, Ireland) was inserted into the costal diaphragm for the continuous measurement of EMG activity. In a similar manner, a concentric needle electrode was inserted into the third intercostal space (T3) for the measurement of external intercostal EMG activity. EMG signals were amplified (×5000), band-pass filtered (50–5000 Hz) and integrated (50 ms time constant; Neurolog system, Digitimer Ltd., Welwyn Garden City, UK). All biological signals were passed through an analogue-to-digital converter (Powerlab r8/30; ADInstruments, Colorado Springs, CO, USA) and were acquired using LabChart 7 (ADInstruments) at a sampling frequency of 4 kHz.

Experimental protocol: Following instrumentation, animals were allowed a period of stabilization for at least 10 min, prior to a 10 min baseline recording. Next, the trachea was occluded for 30–40 s until a plateau was observed in the inspiratory pressure recordings. Following this challenge, animals were allowed to recover and were then instrumented for the measurement of ETCO_2_ and tracheal airflow. Next, animals were vagotomized by bilateral section of the cervical vagi. Following vagotomy, respiratory parameters were recorded for 10 min under steady-state conditions following vagotomy. Next, animals were challenged with hypercapnic-hypoxia (15% O_2_ & 6% CO_2_; 3 min) to examine the effects of chemostimulation on tracheal airflow. Following the experimental protocol, mice were euthanized by decapitation. Blood was collected in 1.5 mL Eppendorf tubes coated with 4% EDTA, which were centrifuged at 1610 *g* for 10 min at 4 °C. Plasma samples were aliquoted and snap frozen in liquid nitrogen and stored at −80 °C. Tibia length and mass were examined as an index of somatic growth. The following organs were weighed (wet weight): spleen, kidney, lung, right heart ventricle and left heart ventricle and interscapular brown adipose tissue and epididymal white adipose tissue.

Data analysis: Integrated inspiratory diaphragm and T3 external intercostal (EIC) EMG activity and peak inspiratory sub-atmospheric oesophageal pressure were analysed. The amplitudes of diaphragm and EIC EMG and oesophageal pressure were averaged under steady-state basal conditions and averaged for the 5 successive maximal sustained efforts (maximal response) of the airway occlusion challenge. *V*_T_ was derived from tracheal airflow measurements following vagotomy during baseline and during hypercapnic-hypoxia. Data for oesophageal pressure and respiratory muscle EMGs in wild type and *mdx* mice were previously published in a separate report [13].

### 2.5. Ex Vivo Diaphragm Muscle Function

The diaphragm muscle (rib and central tendon intact) was immediately excised and placed in a tissue bath at room temperature containing continuously gassed hyperoxic (95% O_2_/5% CO_2_) Krebs solution (in mM: NaCl, 120; KCl, 5; Ca^2+^ gluconate, 2.5; MgSO_4_, 1.2; NaH_2_PO_4_, 1.2; NaHCO_3_, 25; and glucose, 11.5) and *d*-tubocurarine (25 μM) prior to functional analysis. Diaphragm muscle preparations were suspended vertically between two platinum plate electrodes in a water-jacketed tissue bath at 35 °C containing Krebs solution and were continuously aerated with hyperoxia (95% O_2_ & 5% CO_2_). The rib was sutured to an immobile hook and remained in a fixed positon for the duration of the experiment. Using non-elastic string, the central tendon was attached to a lever connected to a dual-mode force transducer (Aurora Scientific Inc.; Aurora, ON, Canada). To determine muscle optimum length (L_o_), the length of the muscle preparations were adjusted using a micro-positioner between intermittent twitch contractions [32,33]. The muscle length which revealed maximal isometric twitch force for a single isometric twitch stimulation (supramaximal stimulation, 1 ms duration) was considered L_o_. Diaphragm preparations were maintained at L_o_ for the duration of the protocol.

Experimental protocol: First, a single isometric twitch contraction was measured and peak isometric twitch force (P_t_), contraction time (CT) and half-relaxation time (½ RT) were assessed. To examine the force-frequency relationship, muscle bundles were stimulated sequentially at 10, 20, 40, 60, 80, 100, 120, 140 and 160 Hz (300 ms train duration). Contractions were interspersed by a 1 min interval. Following the isometric protocol, an isotonic contraction was elicited in preparations at 0% load to examine maximum unloaded muscle shortening and velocity of shortening [32,33].

Data analysis: Muscle bundle cross-sectional area (CSA) was determined for the purpose of normalizing muscle force to bundle size. CSA was calculated by dividing muscle mass (weight in grams) by the product of muscle L_o_ (cm) and muscle density (assumed to be 1.06 g cm^−3^). Muscle force was divided by bundle CSA and expressed as specific force (N cm^−2^). CT and ½ RT were measured as indices of isometric twitch kinetics and expressed in ms. For isotonic contractions, total muscle shortening was considered the total distance shortened during muscle contraction at 0% load. For total muscle shortening (S_max_), data were expressed in absolute units (cm) and were further normalized to L_o_ and expressed in L L_o_^−1^. For the measurement of muscle shortening velocity (V_max_), the distance shortened during the initial 30 ms of an unloaded contraction was assessed [25,33,34] and determined in absolute units (cm s^−1^) and was normalized to L_o_ and expressed in L_o_ s^−1^.

### 2.6. Muscle Immunofluorescence and Histology

#### 2.6.1. Tissue Preparation

Sections of hemidiaphragm from wild type (*n* = 6), *mdx* (*n* = 7) and *mdx +* NAC-treated mice (*n* = 4) were mounted on cubes of liver. Diaphragm samples were embedded in optimum cutting temperature embedding medium (OCT; VWR International, Dublin, Ireland) for cryoprotection and then frozen in isopentane (Sigma Aldrich, Wicklow, Ireland) cooled on dry ice. Samples were then stored at −80 °C for subsequent structural analysis. Serial transverse muscle sections (10 µm) were cut using a cyrostat (Leica CM3050; Leica Microsystems, Nussloch, Germany) at −22 °C and mounted across polylysine-coated glass slides (VWR International, Dublin, Ireland) allowing for a distribution of tissue on a given slide.

#### 2.6.2. Histological Analysis

To examine putative inflammatory cell infiltration of muscle fibres, tissue sections were stained with haematoxylin and eosin (H&E) using an autostainer (Leica ST5010 Autostainer XL, Leica Microsystems, Nussloch, Germany). For collagen staining, picro-sirius red (Leica Biosystems, Wetzlar, Germany) staining was completed. Slides were mounted using DPX mounting medium (Sigma-Aldrich, Wicklow, Ireland), air-dried and visualized on a bright field microscope (Olympus BX51) at ×10 magnification.

Data analysis: A total of six tissue sections per animal were examined. Muscle histology was scored using ImageJ software. Putative inflammatory cell infiltration (the presence of cells in the extracellular matrix) was scored and expressed as a percentage of the total area of muscle. For slides stained with picro-sirius red, the microscope lighting exposure was standardized during imaging. Images were analysed using a colour balance threshold and the area of collagen was expressed as a percentage of the total area of muscle. Data generated from multiple images was averaged per animal before computing group means.

#### 2.6.3. Embryonic Myosin Heavy Chain and Laminin Immunofluorescence

Embryonic myosin (MyHC_EMB_) immunofluorescence was carried out as a marker of fibre regeneration/remodelling [35]. Laminin immunofluorescence was used to examine the fibre size (minimum Feret’s diameter) distribution of the diaphragm muscle as well as to determine central nucleation of muscle fibres. Two slides were removed from the −80 °C freezer containing a minimum of four sections per slide, from two distinct regions of muscle tissue for each animal. A hydrophobic barrier was created around the individual tissue samples using a hydrophobic pen (ImmEdge TM Vector Labs, Peterborough, UK). Briefly, tissue samples were immersed in phosphate-buffered saline (PBS) (0.01 M) containing 1% bovine serum albumin (BSA) for 15 min followed by 3 × 5 min PBS rinses. This was followed by a 30 min wash in PBS containing 5% goat serum (Sigma-Aldrich, Wicklow, Ireland). Before application of the primary embryonic mouse monoclonal antibody (developed by S. Schiaffino and obtained from the Developmental Studies Hybridoma Bank (DSHB) at the University of Iowa, IA, USA), slides were exposed to a mouse IgG blocker, M.O.M. blocking solution (Vector Lab Inc., Burlingame, CA, USA) for 1 h at room temperature. Following 3 × 5 min PBS washes, a cocktail of primary antibodies containing MyHC_EMB_ (F1.652; 1:20) and rabbit anti-laminin antibody (Sigma Aldrich, 1:500) diluted in 1% BSA in PBS was applied to the slides and incubated at 4 °C overnight in a humidity chamber. The next day, slides were washed for 3 × 5 min in PBS before the application of the corresponding secondary antibodies, AlexaFluor594-conjugated goat anti-mouse IgG_1_ (1:500, Jackson ImmunoResearch Ltd., Ely, UK) and fluorescein isothiocyanate (FITC)-conjugated goat anti-rabbit secondary antibody (1:250, Sigma Aldrich). Slides were incubated for 1 h in the dark at room temperature. Finally, slides were washed for 3 × 5 min rinses in PBS before the application of 4′,6-diamidino-2-phenylindole (DAPI; 1:4000, Sigma Aldrich) for 10 min to identify myonuclei, followed by a final 3 × 5 min rinse in PBS. Negative control experiments were run in parallel where the primary antibodies were omitted and the secondary antibodies were applied to ensure that immune-tagging was specific.

*Data analysis:* For muscle fibre distribution, MyHC_EMB_ expressing muscle fibres and central nucleation, muscle sections were viewed at ×10 magnification and images were captured using an Olympus BX51 microscope and an Olympus DP71 camera. Cell Sens™ (Olympus) was used to digitally capture the images. Images were taken at random areas across each muscle section with four muscle sections analysed per animal. Individual images of the laminin, DAPI and MyHC_EMB_ fibres were captured and merged together using ImageJ software. A 600 μm × 600 μm grid with inclusion and exclusion borders was placed over the image for analysis [12]. From each muscle section, the number of fibres per area along with minimum Feret’s diameter of the individual muscle fibres was analysed using a specialized ImageJ macro [36]. Frequency histograms were constructed to illustrate the distribution of fibre size in muscle sections across groups in addition to the coefficient of variation of minimum Feret’s diameter. The number of centrally nucleated and MyHC_EMB_ expressing muscle fibres were counted (using the ImageJ multi-point tool) and expressed relative to the total number of myofibres per recorded area. Data generated from multiple images were averaged per animal before computing group means.

### 2.7. Assays

#### 2.7.1. Tissue Preparation

Frozen diaphragm muscle samples were removed from storage at −80 °C, weighed and homogenized on ice in ice-cold modified radioimmunoprecipitation assay (1X RIPA) buffer, deionized H_2_O, 200 mM sodium fluoride (NaF), 100 mM phenylmethylsulfonylfluoride (PMSF), 1X protease cocktail inhibitor (Sigma Aldrich, Ireland) and 200 mM sodium orthovanadate (Na_ortho_) at a 5% *w*/*v* ratio using a general laboratory homogenizer (Omni-Inc., Kennesaw, GA, USA). Homogenates were allowed 20 min of lyse time on ice with intermittent vortexing. Homogenates were centrifuged in a U-320R centrifuge (Boeckel & Co., Hamburg, Germany) at 15,366 *g* at 4 °C for 20 min to separate insoluble cellular fractions from protein homogenates. The protein-containing supernatant was removed and stored at −80 °C, and the pellet was discarded. The protein concentration of each sample was quantified using a bicinchoninic acid (BCA) assay (Pierce Biotechnology, Fisher Scientific, Dublin, Ireland) as per the manufacturer’s instructions, at a dilution of 1:10.

#### 2.7.2. Glutathione Reductase and Glutathione Peroxidase Activities, Total Glutathione (tGSH) Concentration and GSSG:GSH Ratio

Glutathione reductase (GR) catalyzes the reduction of glutathione disulfide (GSSG) by nicotinamide adenine dinucleotide phosphate (NADPH) to produce reduced glutathione (GSH). GR activity was measured in diaphragm muscle homogenates from all three groups: WT (*n* = 7), *mdx* (*n* = 7) and *mdx* + NAC (*n* = 7). Briefly, 50 μL (25 μg) of sample, 50 μL of KH_2_PO_4_ (0.4 M)/EDTA (4 mM; pH 7.5) buffer and 50 μL of oxidised GSSG (4 mM) was added in duplicate to wells of a clear 96-well plate. Controls were also plated in duplicate which included: (1) all assay components except oxidised GSSG and (2) all assay components except sample, to account for any non-specific reduction in samples tested. 50 μL of NADPH (0.4 mM) was quickly added to each well to initiate the reaction and the absorbance was measured at 340 nM every 15 s for 30 min at 37 °C. The oxidation of NADPH at this wavelength is reflected by a decrease in absorbance over time, proportional to GSSG reduction and thus GR activity. Data are expressed as mU mg^−1^ protein. Glutathione peroxidase (GPx) converts GSH to GSSG while also reducing lipid hyperoxides to alcohols, and hydrogen peroxide (H_2_O_2_) to water. GPx activity was measured in diaphragm muscle homogenates from WT (*n* = 7), *mdx* (*n* = 7) and *mdx* + NAC (*n* = 7) groups using a commercially available kit (catalogue number. ABE3639; Source BioScience, Nottingham, UK) as per the manufacturer’s instructions. Data are expressed as nmol min^−1^ mg^−1^ protein. Total glutathione (tGSH) comprises GSH + GSSG, which can be used to calculate the GSSG:GSH ratio, a common measure of oxidative stress. tGSH and GSSG concentration were measured in diaphragm muscle homogenates from wild type (*n* = 7), *mdx* (*n* = 7) and *mdx* + NAC (*n* = 7) groups using a commercially available kit (catalogue number. 703002; Cayman Chemical, Ann Arbor, MI, USA) as per the manufacturer’s instructions, with data expressed as μM mg^−1^ protein and GSSG:GSH.

#### 2.7.3. Cytokine Multiplex Assay

A multiplex cytokine assay (K15048G-1; Meso Scale Discovery, Rockville, MD, USA) was used to examine cytokine concentrations in diaphragm muscle (200 µg well^−1^; *n* = 7 per group) and plasma samples (1:2 dilution; *n* = 8–10 per group) from all 3 groups. The assay was performed according to the manufacturer’s instructions using an extended incubation time to improve the detection of cytokines in diaphragm muscle homogenates (the plate was incubated overnight at 4 °C) [17]. Following incubation, the plate was read on a Quickplex SQ 120 imager (Meso Scale Discovery).

### 2.8. Gene Expression

#### 2.8.1. RNA Extraction and Preparation

Diaphragm muscle samples were removed from storage at −80 °C, weighed and samples ranging from 20–50 mg were homogenized on ice in Tripure Isolation Reagent (Roche Diagnostics, Ltd., Burgess Hill, UK) using a general laboratory homogenizer (Omni-Inc., Kennesaw, GA, USA). Total RNA was isolated from homogenates in accordance with the manufacturer’s instructions, with an additional chloroform wash step performed during phase separation. The quantity (ng μL^−1^) and purity of isolated RNA (260:280 & 260:230 ratios) was assessed using a Nanodrop 1000 (Thermo Scientific, Waltham, MA, USA) and spectrophotometry. The integrity of isolated RNA was assessed by visualization of distinct 18S and 28S ribosomal RNA bands using an agarose gel electrophoresis system (E-gel, Life Technologies, Carlsbad, CA, USA).

#### 2.8.2. Reverse Transcription

Diaphragm muscle RNA was reverse transcribed to cDNA using a Transcriptor First Strand cDNA Synthesis Kit (Roche Diagnostics Ltd., Burgess Hill, UK) in accordance with the manufacturer’s instructions.

#### 2.8.3. qRT-PCR

cDNA was amplified using Realtime ready Catalog or Custom assays (Roche Diagnostics Ltd., Burgess Hill, UK) shown in Table 1 and Fast Start Essential Probe Master (Roche Diagnostics Ltd., Burgess Hill, UK) as per the manufacturer’s instructions. Briefly, all reactions were carried out in duplicate on a 96-well plate and consisted of 5 µL cDNA and 15 µL master mix using the Lightcycler 96 (Roche Diagnostics Ltd., Burgess Hill, UK). RNA negatives, reverse transcription negatives, cDNA negatives (no template controls) and plate calibrators were used on every plate. Quantification cycle values (Cq) obtained from experiments for the genes of interest (*Nfkb1*, *Fbxo32*, *Trim63*, *Map1lc3b*, *Gabarapl1*, *Bnip3*, *Cybb*, *Park2*, *Pink*, *Nfe2l2*, *Cat*, *Mstn*, *Myod1*, *Gpx2*, *Myog*, *Sod2*, *Sod1*, *Sirt1*, *Igf1 and Mef2c*) were normalized to that of a reference gene, *Hprt1*, to account for variations in input amounts of RNA/cDNA and the efficiency of reverse transcription. *Hprt1* was found to be the most stable gene of the candidate reference genes screened, in consideration of genotype and drug treatment (NAC). The relative gene expression was calculated using the ΔΔCT method (normalized expression of the gene of interest to that of the reference gene), with a change in expression shown as a fold change relative to the control group (wild type).

### 2.9. Statistical Analysis

Data are expressed as mean ± SD or as box and whisker plots (median, 25–75th percentile and scatter plot). Using Prism, version 6.0 (Graphpad Software Inc., San Diego, CA, USA), data from each group were statistically analysed and assessed for normal distribution and equal variances. Data sets that were of equal variance and normally distributed were compared statistically using one-way analysis of variance (ANOVA) with a Newman–Keuls *post hoc* test. Kruskal-Wallis with Dunn’s *post hoc* test was applied to group data that were not of Gaussian distribution. Diaphragm muscle force-frequency relationship was statistically analysed using repeated measures two-way ANOVA with a Bonferroni *post hoc* test. *P* values are reported.

## 3. Results

### 3.1. Body Mass and Organ Measurements

Table 2 compares body mass, tibia length and mass, organ mass and brown and white adipose mass in wild type, *mdx* and *mdx* mice treated with 1% NAC (*mdx* + NAC). Body mass for *mdx* mice was unaffected by NAC supplementation (*P* > 0.05; Kruskal-Wallis with Dunn’s *post hoc* test). Right and left ventricle mass was increased for *mdx* mice (*P* < 0.0001 and *P* < 0.01; one-way ANOVA with Newman-Keuls *post hoc* test and Kruskal–Wallis with Dunn’s *post hoc* test; right and left ventricle, respectively). The ratio of right to left ventricle mass (RV:LV) was increased in *mdx* (*P* < 0.05; one-way ANOVA with Newman–Keuls *post hoc* test); RV:LV was unaffected by NAC. Spleen mass was increased in *mdx* (*P* < 0.0001) compared with wild type and was further increased in *mdx* mice following NAC intervention (*P* < 0.01). Brown and white adipose tissue mass were lower in *mdx* (*P* < 0.01 and *P* < 0.001; one-way ANOVA with Newman–Keuls *post hoc* test and Kruskal-Wallis with Dunn’s *post hoc* test; brown and white, respectively) compared with wild type. Brown and white adipose tissue mass was unchanged by NAC administration. Tibia length and mass, and kidney and lung mass were equivalent in all three groups (Table 2).

### 3.2. Baseline Ventilation and Ventilatory Responsiveness to Hypercapnic-Hypoxia in Conscious Mice

Respiratory parameters including *f*_R_, *V*_T_ and *V*_E_ during baseline (21% O_2_) and hypercapnic-hypoxia (10% O_2_ & 6% CO_2_) are shown in Table 3. During baseline conditions, *f*_R_, *V*_T_ and *V*_E_ were equivalent between all three groups. During exposure to hypercapnic-hypoxia, *V*_T_ was lower in *mdx* (*P* < 0.05; Kruskal–Wallis with Dunn’s *post hoc* test) compared with wild type, *f*_R_ and *V*_E_ were equivalent between wild type and *mdx*. NAC did not alter *f*_R_, *V*_T_ and *V*_E_ during hypercapnic-hypoxia.

### 3.3. Inspiratory Pressure and Diaphragm and Intercostal EMGs during Airway Obstruction in Anaesthetized Mice

Previously, we reported findings from recordings of oesophageal pressure and diaphragm and external intercostal EMG activities for two of the groups, wild type and *mdx*, in the context of a comprehensive assessment of respiratory system deficits and compensations in young *mdx* mice [13]. Here, we compare values for the *mdx* + NAC group to our previously published values. During baseline recordings, oesophageal pressure (*P* = 0.9843; Kruskal–Wallis test; Table 4) and diaphragm (*P* = 0.0907; Kruskal-Wallis test; Table 4) and external intercostal (*P* > 0.05; one-way ANOVA with Newman-Keuls *post hoc* test; Table 4) EMG activities in *mdx* + NAC were equivalent to values for *mdx*. In anaesthetized mice, protracted airway obstruction increased oesophageal sub-atmospheric pressure generation and EMG activity in all groups, measured as the average of five consecutive peak inspiratory efforts. Compared with *mdx* mice, oesophageal pressure (*P* = 0.0954; one-way ANOVA; Table 4) and diaphragm (*P* = 0.1741; Table 4) and external intercostal (*P* > 0.05; Kruskal–Wallis with Dunn’s *post hoc* test; Table 4) EMG activities were not different following NAC administration during protracted airway occlusion.

### 3.4. Tracheal Airflow in Anaesthetized Mice

Table 5 shows respiratory data in anaesthetized wild type, *mdx* and *mdx* + NAC mice during baseline conditions (60% O_2_) and hypercapnic-hypoxia (F_i_O_2_ = 0.15 & F_i_CO_2_ = 0.05) in vagotomized mice. All respiratory parameters were equivalent comparing wild type, *mdx* and *mdx* + NAC mice.

### 3.5. Diaphragm Muscle Contractile Function Ex Vivo

Table 6 shows data for twitch contractile kinetics (CT and ½ RT) and isotonic contractile kinetics (S_max_ and V_max_) of diaphragm muscle preparations from all three groups. Diaphragm CT and ½ RT were equivalent in wild type and *mdx* preparations; NAC had no effect on CT and ½ RT for *mdx* diaphragm. Absolute values for S_max_ were reduced in *mdx* (*P* < 0.01; Kruskal-Wallis with Dunn’s *post hoc* test) diaphragm preparations compared with wild type. NAC supplementation in *mdx* mice increased diaphragm S_max_ and V_max_ (*P* < 0.05 and *P* < 0.05, S_max_ and V_max_ respectively; Kruskal-Wallis with Dunn’s *post hoc* test). Normalized values for S_max_ and V_max_ were equivalent in all three groups.

**Figure 1:**
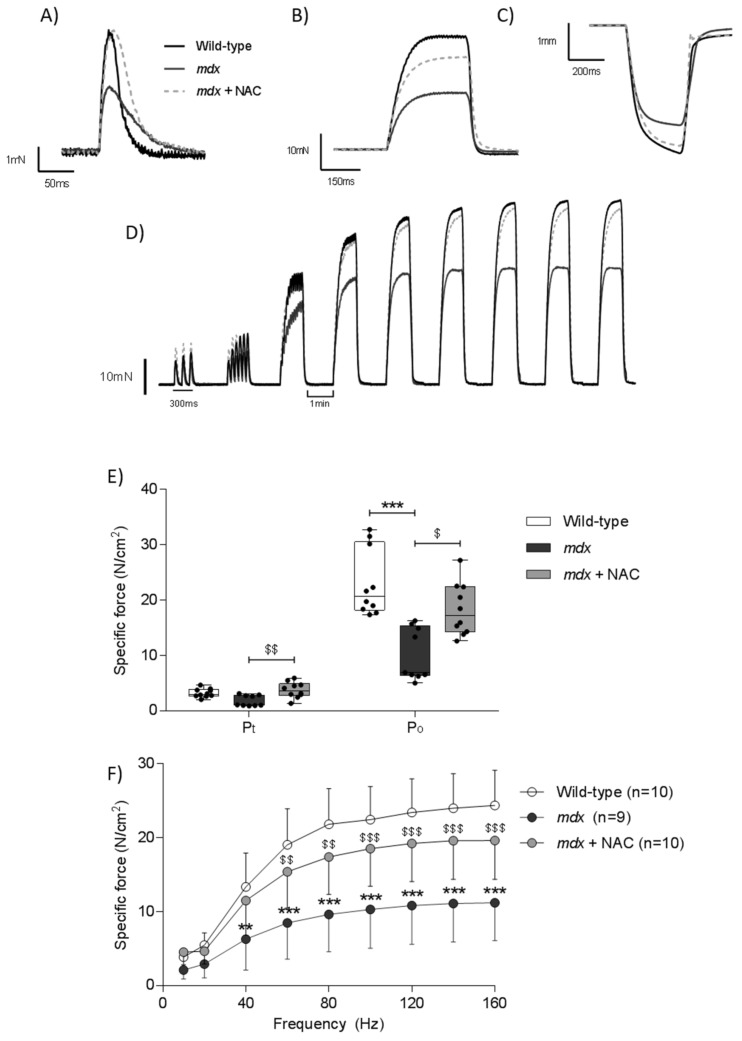
Ex vivo diaphragm muscle contractile force. A–D, original traces of ex vivo diaphragm muscle twitch force (**A**), tetanic force at 100Hz (**B**), maximum unloaded shortening (**C**) and force-frequency relationship (**D**) for wild type (WT), *mdx* and *mdx* + N-acetylcysteine (NAC) preparations. E and F, group data for ex vivo diaphragm muscle twitch (**E**; P_t_) and tetanic (**E**; P_o_) force and force-frequency relationship (**F**) in wild type, *mdx* and *mdx* + NAC preparations. *Mdx* + NAC group received 1% N-acetylcysteine in the drinking water for 14 days. For P_t_ and P_o_, values are expressed as scatter point box and whisker plots (median, 25–75 percentile and scatter plot) and were statistically compared by Kruskal-Wallis with Dunn’s *post hoc* test. For force-frequency, data are shown as mean ± SD and were statistically compared using repeated measures two-way ANOVA with Bonferroni *post hoc* test. * denotes *mdx* different from corresponding WT group; ** *P* < 0.01, *** *P* < 0.001. ^$^ denotes *mdx* + NAC different from corresponding *mdx* group; ^$^ *P* < 0.05, ^$$^ *P* < 0.01, ^$$$^ *P* < 0.001.

Figure 1 shows representative original traces for diaphragm muscle twitch (A) and tetanic (B) contractions, force-frequency relationship (C) and maximum unloaded shortening (D). NAC treatment increased diaphragm twitch force in *mdx* (Figure 1E, P_t_; *P* < 0.01; Kruskal–Wallis with Dunn’s *post hoc* test) compared with *mdx*. Diaphragm tetanic force at 100 Hz was lower in *mdx* (Figure 1E, P_o_; *P* < 0.001; Kruskal–Wallis with Dunn’s *post hoc* test) compared with wild type. NAC treatment increased *mdx* diaphragm tetanic force (Figure 1E, P_o_; *P* < 0.05; Kruskal–Wallis with Dunn’s *post hoc* test) compared with *mdx*. For the force-frequency relationship, diaphragm specific force was reduced in *mdx* compared with wild type preparations (Figure 1F; *P* < 0.0001; repeated measures two-way ANOVA). *Post hoc* analysis revealed differences between wild type and *mdx* diaphragm across a broad stimulus range (40–160 Hz). NAC supplementation to *mdx* mice improved diaphragm force-generating capacity compared with *mdx* preparations (Figure 1F; *P* < 0.0001; repeated measures two-way ANOVA). *Post hoc* analysis indicated an improvement in *mdx* diaphragm force-generating capacity following NAC supplementation across a broad stimulus range (60–160 Hz).

### 3.6. Diaphragm Muscle Histology and Immunofluorescence

**Figure 2:**
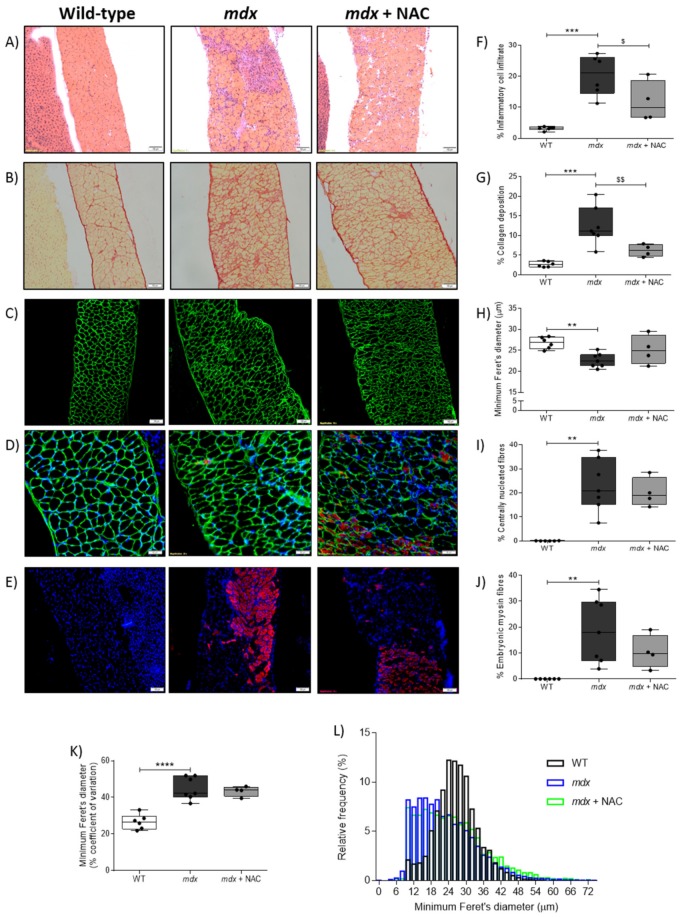
Diaphragm muscle histology and immunofluorescence. A and B, representative histological images of transverse sections of diaphragm muscle stained with Haematoxylin and Eosin (**A**) and Sirius red (**B**) in wild type (WT), *mdx* and *mdx* + NAC groups. *Mdx* + NAC group received 1% N-acetylcysteine in the drinking water for 14 days. C-E, representative images of diaphragm muscle immunofluorescently labelled for laminin (**C**), laminin, DAPI and embryonic myosin (**D**) and embryonic myosin and DAPI (**E**) for WT, *mdx* and *mdx* + NAC groups. Scale bars represent 100 μm in A, B, C and E. In D, scale bars represent 50 μm. F–J, group data showing the relative area of infiltration of inflammatory cells (**F**), relative area of collagen deposition (**G**), muscle fibre size measured by minimum Feret’s diameter (**H**), relative area of centrally nucleated diaphragm muscle fibres (**I**) and relative area of diaphragm muscle fibres expressing embryonic myosin (**J**) in diaphragm muscle from WT, *mdx* and *mdx* + NAC groups. K-L, group data for the coefficient of variation of muscle fibre size (**K**) and the frequency distribution of muscle fibre size (**L**) for all three groups. Values are expressed as scatter point box and whisker plots (median, 25–75 percentile and scatter plot) and were statistically compared using one-way ANOVA with Newman-Keuls *post hoc* test, or Kruskal-Wallis with Dunn’s *post hoc* test. * denotes *mdx* different from corresponding WT group; ** *P* < 0.01, *** *P* < 0.001 and **** *P* < 0.0001. ^$^ denotes *mdx* + NAC different from corresponding *mdx* group; ^$^ *P* < 0.05 and ^$$^ *P* < 0.01.

Figure 2A–E shows representative images for diaphragm muscle histology and immunohistochemistry for all three groups. The areal density of inflammatory cell infiltration was increased in *mdx* diaphragm compared with wild type (Figure 2F; *P* < 0.001; one-way ANOVA with Newman–Keuls *post hoc* test). There was a reduction in immune cell infiltration in *mdx* diaphragm following NAC treatment compared with *mdx* diaphragm (Figure 2F; *P* < 0.05). The areal density of collagen labelled with Sirius red staining was increased in *mdx* diaphragm compared with wild type (Figure 2G; *P* < 0.001). NAC treatment in *mdx* resulted in a reduction in collagen deposition compared with untreated *mdx* mice (Figure 2G; *P* < 0.01). There was a reduction in muscle fibre size (minimum Feret’s diameter) for *mdx* diaphragm compared with wild type (Figure 2H; *P* < 0.01); NAC treatment had no effect on fibre size. The proportion of diaphragm muscle fibres with centrally located myonuclei was increased in *mdx* compared with wild type (Figure 2I; *P* < 0.01; Kruskal–Wallis with Dunn’s *post hoc* test); NAC had no effect on the proportion of centrally located myonuclei. The proportion of diaphragm muscle fibres expressing embryonic myosin was increased in *mdx* diaphragm compared with wild type (Figure 2J; *P* < 0.01; one-way ANOVA with Newman–Keuls *post hoc* test); NAC treatment had no effect on central nucleation and embryonic myosin expression in *mdx* diaphragm. The coefficient of variation of muscle fibre size was increased in *mdx* diaphragm compared with wild type (Figure 2K; *P* < 0.0001); NAC treatment had no effect on fibre size variability. There was a wider distribution of muscle fibre size (based on minimum Feret’s diameter) in *mdx* diaphragm compared with wild type (Figure 2L). The distribution of diaphragm muscle fibres in *mdx* + NAC was similar to *mdx* mice, with a slightly greater abundance of large fibres.

### 3.7. Diaphragm Glutathione Status

**Figure 3:**
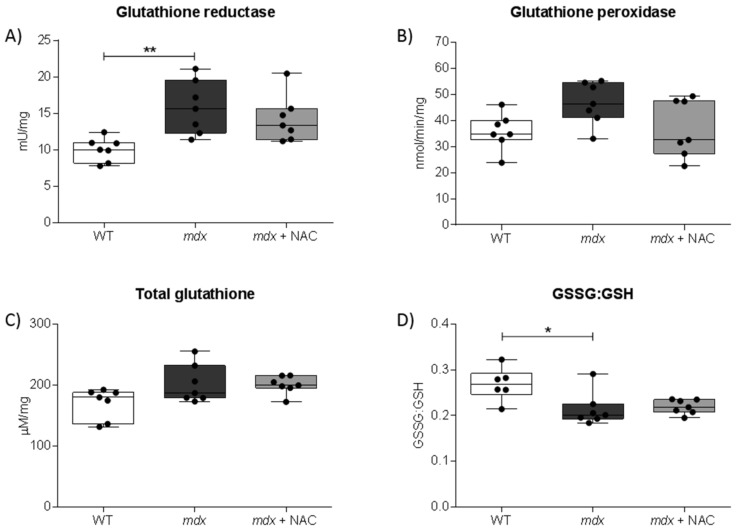
Diaphragm muscle glutathione status. A and B, group data for glutathione reductase (**A**) and glutathione peroxidase (**B**) activities of diaphragm muscle from wild type (WT), *mdx* and *mdx* + NAC groups. *Mdx* + NAC group received 1% N-acetylcysteine in the drinking water for 14 days. C and D, group data for total glutathione (**C**) and the ratio of oxidised glutathione (GSSG) to glutathione (GSSG:GSH; **D**). Values are expressed as scatter point box and whisker plots (median, 25–75 percentile and scatter plot) and were statistically compared using one-way ANOVA with Newman-Keuls *post hoc* test, or Kruskal–Wallis with Dunn’s *post hoc* test. * denotes *mdx* different from corresponding WT group; * *P* < 0.05 and ** *P* < 0.01.

Glutathione reductase activity was increased in *mdx* diaphragm (Figure 3A; *P* < 0.01; one-way ANOVA with Newman-Keuls *post hoc* test) compared with wild type. GSSG:GSH was decreased in *mdx* diaphragm (Figure 3D; *P* < 0.05; Kruskal-Wallis with Dunn’s *post hoc* test) compared with wild type. Diaphragm glutathione peroxidase and total glutathione were equivalent in all three groups. Diaphragm glutathione reductase, glutathione peroxidase, total glutathione and GSSG:GSH were all unaffected by NAC supplementation in *mdx* mice.

### 3.8. Plasma and Diaphragm Cytokine Concentrations

**Figure 4:**
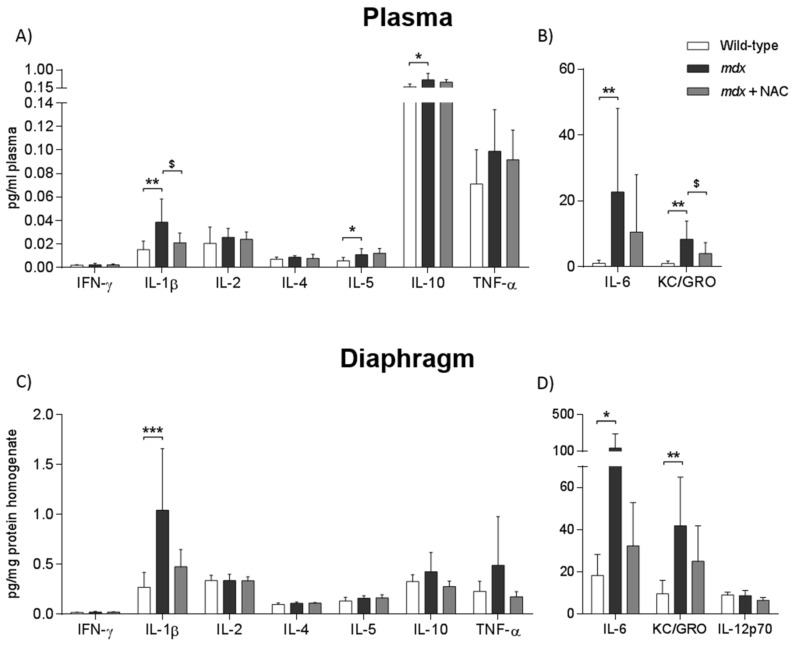
Plasma and diaphragm muscle cytokine concentrations. A–D, group data for selective cytokine concentrations in plasma and diaphragm muscle from wild type (*n* = 7), *mdx* (*n* = 7–9) and *mdx* + NAC (*n* = 7–9) groups. *Mdx* + NAC group received 1% N-acetylcysteine in the drinking water for 14 days. Plasma cytokines include interferon-γ (IFN-γ; **A**), interleukin-1β (IL-1β; **A**), interleukin-2 (IL-2; **A**), interleukin-4 (IL-4; **A**), interleukin-5 (IL-5; **A**), interleukin-6 (IL-6; **B**), interleukin-10 (IL-10; **A**), KC/GRO (**B**), tumour necrosis factor-α (TNF-α; A). Diaphragm cytokines include IFN-γ (**C**), IL-1β (**C**), IL-2 (**C**), IL-4 (**C**), IL-5 (**C**), IL-6 (**D**), IL-10 (P), KC/GRO (**D**), IL-12 (IL-12p70; **D**) and TNF-α (**C**). Data are shown as mean ± SD and were statistically compared using one-way ANOVA with Newman–Keuls *post hoc* test, or Kruskal–Wallis with Dunn’s *post hoc* test. * denotes *mdx* different from corresponding WT group; * *P* < 0.05, ** *P* < 0.01 and ***P < 0.001. ^$^ denotes *mdx* + NAC different from corresponding *mdx* group; ^$^ *P* < 0.05.

Plasma samples from *mdx* mice had increased concentration of the cytokines, IL-1β (Figure 4A; *P* < 0.01; one-way ANOVA with Newman–Keuls *post hoc* test), IL-5 (Figure 4A; *P* < 0.05); IL-6 (Figure 4B; *P* < 0.01; Kruskal–Wallis with Dunn’s *post hoc* test) and IL-10 (Figure 4A; *P* < 0.05; Kruskal–Wallis with Dunn’s *post hoc* test). The chemokine KC/GRO was increased in *mdx* plasma (Figure 4B; *P* < 0.01; one-way ANOVA with Newman–Keuls post hoc test) compared with wild type. NAC supplementation decreased the concentration of IL-1β (Figure 4A; *P* < 0.01; one-way ANOVA with Newman–Keuls post hoc test) and KC/GRO (Figure 4B; *P* < 0.05; one-way ANOVA with Newman–Keuls post hoc test) in *mdx* plasma. Plasma IFN-γ (Figure 4A), IL-2 (Figure 4A), IL-4 (Figure 4A) and TNF-α (Figure 4A) concentrations were equivalent in all three groups. Increased levels of IL-1β (Figure 4C; *P* < 0.001; Kruskal-Wallis with Dunn’s *post hoc* test), IL-6 (Figure 4D; *P* < 0.05; Kruskal–Wallis with Dunn’s *post hoc* test) and KC/GRO (Figure 4D; *P* < 0.01; one-way ANOVA with Newman–Keuls post hoc test) were observed in *mdx* diaphragm. *Post hoc* analysis revealed no changes in *mdx* diaphragm IL-1β, IL-6 or KC/GRO concentrations following NAC (P >0.05) but notably the concentrations of IL-1β and IL-6 in diaphragms from *mdx* + NAC were >50% reduced compared with *mdx*. Diaphragm IFN-γ (Figure 4C), IL-2 (Figure 4C), IL-4 (Figure 4C), IL-5 (Figure 4C), IL-10 (Figure 4C), TNF-α (Figure 4C) and IL-12p70 (Figure 4D) were equivalent in all three groups.

### 3.9. Diaphragm Muscle mRNA Expression

**Figure 5:**
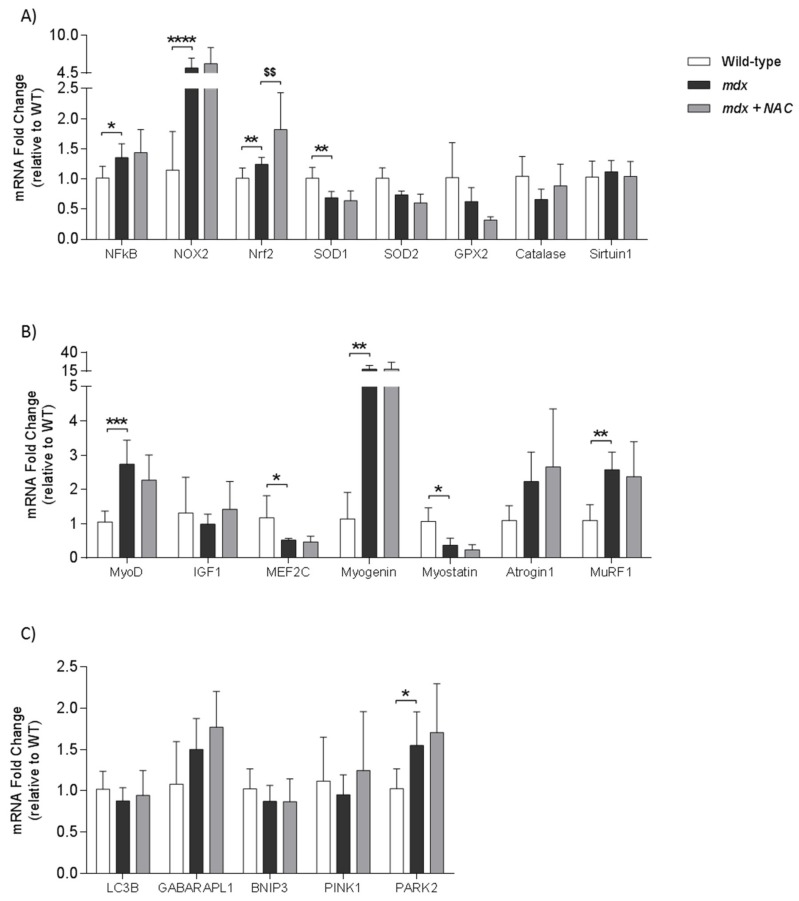
Diaphragm muscle mRNA expression. *Definition of abbreviations:* BNIP-3, BCL3 interacting protein; GPX, glutathione peroxidase; GABARAPL1, GABA type A receptor associated protein Like 1; IGF, insulin-like growth factor; LC3B, light chain 3 beta; MEF2C, myocyte enhancer factor 2C; MyoD, myogenic differentiation; Murf-1, muscle RING-finger protein-1; NAC, N-acetylcysteine; NOX, NADPH oxidase; NRf-2, nuclear factor (erythroid-derived 2)-like 2; NFκB, nuclear factor kappa-light-chain-enhancer of activated B cells; PARK-2, parkin RBR E3 ubiquitin protein ligase; PINK-1, phosphatase and tensin homolog (PTEN)-induced kinase 1; SOD, superoxide dismutase; WT, wild type. A–C, group data for mRNA expression of NFκB (**A**), NOX2 (**A**), Nrf2 (**A**), SOD1 (**A**), SOD2 (**A**), GPX2 (**A**), catalase (**A**), sirtuin1 (**A**), MyoD (**B**), IGF1 (**B**), MEF2C (**B**), myogenin (**B**), myostatin (**B**), atrogin1 (**B**), MuRF1 (**B**), LC3B (**C**), GABARAPL1 (**C**), BNIP3 (**C**), PINK1 (**C**) and PARK2 (**C**) in diaphragm muscle from wild type (WT; *n* = 7–8), *mdx* (*n* = 6–7) and *mdx* + NAC (*n* = 6–7) groups. *Mdx* + NAC group received 1% N-acetylcysteine in the drinking water for 14 days. Data are shown as mean ± SD and were statistically compared using one-way ANOVA with Newman-Keuls *post hoc* test, or Kruskal-Wallis with Dunn’s *post hoc* test. * denotes *mdx* different from corresponding WT group; * *P* < 0.05, ** *P* < 0.01, *** *P* < 0.001 and **** *P* < 0.0001. ^$^ denotes *mdx* + NAC different from corresponding *mdx* group; ^$$^ *P* < 0.01.

Figure 5 shows data for mRNA expression of genes related to redox status, inflammation, muscle repair, autophagy and mitophagy in diaphragm muscle from wild type, *mdx* and *mdx* + NAC groups. There was increased expression of the pro-inflammatory transcription factor NFκB in *mdx* diaphragm (Figure 5A; *P* = 0.0184; one-way ANOVA with Newman-Keuls *post hoc* test) compared with wild type; NAC administration had no effect. The expression of the reactive oxygen species generating complex NOX2 was increased in *mdx* diaphragm (Figure 5A; *P* < 0.0001) and was unaffected by NAC administration. Expression of the antioxidant response element, Nrf2, was similar in wild type and *mdx* diaphragm (*P* > 0.05); Nrf2 expression was increased in *mdx* + NAC compared with *mdx* (Figure 5A; *P* < 0.01). Expression of the antioxidant gene SOD1 was decreased in *mdx* diaphragm (Figure 5A; *P* < 0.01) compared with wild type; NAC treatment in *mdx* had no effect. There were no differences between groups for SOD2 and GPX2 in *post hoc* analyses.

The muscle repair genes MyoD and Myogenin had increased mRNA expression in *mdx* diaphragm (Figure 5B; *P* < 0.001 and *P* = 0.01; MyoD and Myogenin respectively; one-way ANOVA with Newman-Keuls *post hoc* test and Kruskal–Wallis with Dunn’s *post hoc* test respectively); NAC treatment in *mdx* had no effect on both genes. mRNA expression of Myostatin, an inhibitor of myogensis, was reduced in *mdx* diaphragm (Figure 5B; *P* < 0.01; one-way ANOVA with Newman–Keuls *post hoc* test) compared with wild type diaphragm; NAC treatment in *mdx* had no effect. The muscle differentiation transcription factor MEF2C had reduced mRNA expression in *mdx* diaphragm compared with wild type (Figure 5B; *P* < 0.05) and was unaffected by NAC treatment in *mdx*.

The atrophy-related gene MuRF1 had increased mRNA expression in *mdx* diaphragm (Figure 5B; P < 0.01) compared with wild type; MuRF1 expression was unaffected by NAC administration. The mitophagy-related gene PARK2, had increased mRNA expression in *mdx* diaphragm (Figure 5C; *P* < 0.05) compared with wild type; PARK2 mRNA expression was unaffected by NAC treatment in *mdx*. There were no differences between groups for GABARAPL1 in *post hoc* analyses. mRNA expression of Catalase (Figure 5A), Sirtuin1 (Figure 5A), IGF1 (Figure 5B), Atrogin1 (Figure 5B), PINK1 (Figure 5C), LC3B (Figure 5C) and BNIP3 (Figure 5C) was equivalent in all three groups.

## 4. Discussion

The major findings of the study are: (i) NAC supplementation improved *mdx* diaphragm functional capacity (ii) NAC supplementation reduced collagen deposition (fibrosis) and immune cell infiltration in *mdx* diaphragm; (iii) NAC treatment reduced IL-1β and KC/GRO concentrations in *mdx* plasma samples; (iv) NAC increased Nrf2 mRNA expression in *mdx* diaphragm; (v) NAC had no adverse effect on ventilation, inspiratory pressure and respiratory muscle EMG or growth measures in *mdx* mice; (vi) NAC did not recover decreased respiratory muscle EMG activity in *mdx* mice during maximum activation (airway obstruction).

Dystrophin deficiency in DMD results in mechanical deficits in striated muscle [5]. Respiratory muscle weakness occurs in DMD, with major implications for respiratory control [37]. Inspiratory muscle strength declines with increasing age in DMD boys [6]. Respiratory disturbances are common during sleep, including hypoventilation [38,39] and apnoeic events [37,40,41]. The *mdx* mouse model of DMD has characteristics similar to DMD, including disruptions to normal breathing [12,42,43,44] and diaphragm and pharyngeal dilator muscle weakness [12,15,16,32,45]. Adequate respiratory muscle function is essential for ventilation and mechanical manoeuvres including coughing and sneezing, mechanisms which safeguard against airway collapse. Given the clinical implications of poor respiratory system function in DMD, the development of new therapeutic strategies aimed at boosting respiratory muscle performance is essential.

Herein, diaphragm muscle weakness in *mdx* mice was evidenced by reduced tetanic force, consistent with previous reports in young and older *mdx* mice [12,13,14,16,17]. Specific force was reduced within the frequency range of 40–160 Hz for *mdx* diaphragm compared with wild type. This frequency range corresponds to a broad range of ventilatory and non-ventilatory behaviours [13,46,47,48]. Structural abnormalities occur in dystrophic muscle as a consequence of muscle fibre damage and degeneration. Our study confirmed structural alterations in *mdx* diaphragm, including a substantial increase in the number of centralized myonuclei (measure of fibre regeneration following damage), increased expression of MyHC_EMB_ and increased collagen content (fibrosis). We observed a leftward shift in the frequency distribution of dystrophic muscle fibre size and a concomitant increase in the variability of fibre size. These observations are consistent with and extend our previous findings [12,17] and those of others [49,50], revealing substantial diaphragm muscle pathology in young (8 week old) *mdx* mice.

Systemic and muscle inflammation are prominent features of DMD, secondary to muscle fibre damage. There is elevated expression of chemokines [51,52] and pro-inflammatory cytokines [53,54] in serum and muscle biopsies from DMD boys. In the current study, we report elevated pro-inflammatory (IL-1β and IL-6) and anti-inflammatory (IL-10) cytokine concentrations in plasma samples from *mdx* mice. Immune cells are recruited to areas of damaged muscle, evidenced by increased immune cell infiltration in dystrophic muscle [55]. Here, we report an increase in putative inflammatory cells in *mdx* diaphragm. The concentrations of the pro-inflammatory cytokines, IL-1β and IL-6, and the chemokine KC/GRO are increased in *mdx* diaphragm. Anti-inflammatory drugs have been shown to have beneficial effects on *mdx* skeletal muscle form, but limited improvements in muscle force [55,56]. Encouragingly, however, blockade of pro-inflammatory cytokine signalling has proved beneficial to *mdx* respiratory muscle form and function [17,33,57,58].

Recent studies suggest a close relationship between oxidative stress and inflammation in dystrophic muscle pathology [4,21,59]. Oxidative stress develops as a consequence of increased generation of ROS and/or decreased endogenous antioxidant capacity. Immune cells in muscle can produce ROS [60], which at high levels can lead to impaired muscle force-generating capacity [19]. ROS are primarily produced by the mitochondria and enzyme complexes such as NADPH oxidase (NOX) and xanthine oxidase. Cellular damage occurs during periods of excessive ROS production. Muscle biopsies from DMD boys indicate increased levels of lipid peroxidation, an indirect measure of elevated ROS [61]. Expression of the ROS generating enzyme NOX2 is elevated in DMD muscle [4]. Endogenous antioxidant defence appears to be curtailed in DMD. For example, the activity of superoxide dismutase is decreased in DMD muscle [62]. Studies of the glutathione system in DMD boys suggests glutathione deficiency [63,64]. In DMD muscle biopsies, decreased antioxidant activity confers reduced antioxidant defence increasing the risk of oxidative damage. Similarly, diaphragm muscle weakness in *mdx* mice is associated with altered redox balance [21,65]. ROS production is increased in *mdx* muscle [66,67]. NOX is elevated in *mdx* diaphragm and limb muscle [68,69,70]. Levels of 4-hydroxynonenal are elevated in *mdx* diaphragm, indicating increased lipid peroxidation [28]. Respiratory and limb muscle samples from *mdx* mice have decreased citrate synthase activity, suggesting reduced mitochondrial content [22,27]. Protein thiol oxidation and carbonyl content are increased in *mdx* diaphragm [71], further supporting redox modulation of dystrophic respiratory muscle.

Our examination of diaphragm mRNA expression indicated an increase in mRNA expression related to muscle atrophy (MuRF1), regeneration (MyoD, myogenin), inflammation (NFκB), oxidative stress (NOX2) and mitophagy (PARK2) in *mdx* diaphragm, and a reduction in antioxidant (SOD1)-related mRNA expression compared with wild type. The transcription factor Nrf2 is considered a regulator of antioxidant proteins in response to oxidative injury or inflammation. Nrf2 mRNA was increased in *mdx* diaphragm, consistent with studies of elevated Nrf2 in DMD muscle [4]. An upregulation of Nrf2 signalling in dystrophic muscle suggests a defensive antioxidant response.

We investigated the effects of NAC supplementation in *mdx* mice on ventilation in conscious mice, and inspiratory pressure and respiratory (diaphragm and external intercostal) EMG activity in anaesthetized mice. In addition, we determined diaphragm muscle structure and force-generating capacity, diaphragm glutathione, diaphragm and plasma cytokine concentrations, and diaphragm mRNA expression. NAC is a free radical scavenger and a precursor of reduced glutathione (GSH), boosting synthesis of the endogenous antioxidant. We administered 1% NAC in the drinking water to *mdx* mice for 14 consecutive days beginning at 6 weeks of age. We have previously demonstrated the beneficial effects of NAC supplementation in rodent models of hypoxia-induced respiratory muscle weakness, where NAC improves respiratory muscle force-generating capacity and boosts endogenous cell survival signalling [24,25,26,72]. In addition to its effects on the glutathione system and its important role as a ROS scavenger, NAC is also a mucolytic agent and has been considered as a therapeutic agent in respiratory conditions such as chronic obstructive pulmonary disease [73]. Previously, NAC was shown to attenuate diaphragm muscle fatigue in humans [74].

In the current study, NAC supplementation increased diaphragm specific force in *mdx* mice across the frequency range of 60–160 Hz. Structural improvements following NAC included a reduction in diaphragm collagen content and a decrease in the infiltration of putative inflammatory cells. Total absolute muscle shortening and maximal shortening velocity were both improved in *mdx* diaphragm following NAC supplementation due to improvements in *mdx* muscle optimal length. This was likely mediated, at least in part, by the reduction in diaphragm collagen content following NAC, decreasing tissue stiffness. Recent studies of NAC administration in *mdx* mice demonstrated improved grip strength, increased EDL force output *ex vivo*, and reduced muscle inflammation and oxidative stress [27]. Whitehead et al. (2008) also reported increased EDL force and reduced muscle damage and inflammation following NAC intervention [30]. Furthermore, NAC reduced myosin protein thiol oxidation in *mdx* limb muscle [27]. Interestingly, NAC has been shown to prevent myofibre damage in *mdx* mice following exercise [29,75]. Anti-fibrotic actions of NAC have also been observed in animal models of disease. For example, NAC decreased TGFβ signalling in fibroblasts [76]; cardiac fibrosis was decreased by NAC in a mouse model of heart failure [77]; NAC decreased fibrosis in soleus muscle in a murine model of peripheral arterial insufficiency [78]; and renal fibrosis was decreased by NAC in a model of dilated cardiomyopathy [79].

We examined the glutathione system in the diaphragm of wild type, *mdx* and *mdx* + NAC mice. Glutathione is an endogenous antioxidant that exists in both a reduced (GSH) and oxidized (GSSG) state. Glutathione peroxidase (GPx) catalyses the oxidation reaction of GSH to GSSG, whereas glutathione reductase catalyses the reduction of GSSG to GSH. Glutathione reductase activity was increased in *mdx* diaphragm, along with a reduction in the GSSG:GSH ratio. These data suggest an early antioxidant defence, perhaps in response to a modest oxidative stress (buffered by glutathione reductase) in diaphragm from young *mdx* mice.

Given the close relationship between inflammation and oxidative stress, we examined immune cell infiltration in the diaphragm and the concentration of cytokines in diaphragm and plasma samples. Supplementation with NAC reduced the relative area of infiltrating inflammatory cells in *mdx* diaphragm. Cytokine concentrations were determined in plasma and diaphragm muscle samples following NAC administration. The pro-inflammatory cytokine IL-1β and the chemokine KC/GRO were reduced in *mdx* plasma samples following NAC. In addition, there was a sizeable reduction in plasma IL-6 levels (11 ± 18 *versus* 23 ± 25 pg.ml^−1^ plasma; *mdx* + NAC *versus mdx*). For diaphragm, sizeable reductions in the concentration of IL-1β (0.47 ± 0.17 *versus* 1.04 ± 0.62 pg.mg^−1^ protein homogenate; *mdx* + NAC *versus mdx*), IL-6 (32 ± 21 *versus* 137 ± 155 pg.mg^−1^ protein homogenate) and KC/GRO (25 ± 17 *versus* 42 ± 23 pg.mg^−1^ protein homogenate) were observed following NAC. These data are in agreement with previous studies of NAC intervention in *mdx* mice. Pinniger et al. (2017) demonstrated that NAC supplementation reduced neutrophils and myeloperoxidase activity in *mdx* gastrocnemius muscle [27]. De Senzi Moraes Pinto et al. (2013) reported that NAC supplementation in young *mdx* mice reduced TNF-α protein expression [28]. Given the emerging benefits of reduced pro-inflammatory cytokine signalling in *mdx* respiratory muscle, these anti-inflammatory effects of NAC likely contributed to improved respiratory muscle performance in *mdx* mice. Our study suggests that the potential anti-inflammatory effects of NAC in dystrophic muscle warrant further attention.

Breathing was assessed using whole-body plethysmography in conscious mice during baseline (21% O_2_) and chemoactivated breathing with hypercapnic-hypoxia (F_i_O_2_ = 0.10 and F_i_CO_2_ = 0.06). These assessments confirmed the capacity for *mdx* mice to enhance ventilation as previously described [12,13]. Tidal volume generation during hypercapnic-hypoxia was lower in *mdx* compared with wild type and was not rescued by NAC. In addition, we examined respiratory airflow and volume measures in anaesthetized mice following bilateral section of the vagi and with superimposed chemo-activation (F_i_O_2_ = 0.15 and F_i_CO_2_ = 0.06) to maximally activate ventilation. *V*_T_ and inspiratory and expiratory flows were equivalent or greater in *mdx* mice compared with wild type revealing preserved ventilatory capacity in young *mdx* mice despite dystrophic disease. Direct measures of *f*_R_, *V*_T_ and *V*_E_ and peak inspiratory and expiratory flows were equivalent between *mdx* and *mdx* + NAC groups, revealing no adverse effect of NAC on respiratory airflow and volume in *mdx* mice.

Our recent study reported lower diaphragm EMG activity in anaesthetized *mdx* mice during maximum activation [13]. Despite structural remodelling, profound weakness and lower EMG activity in *mdx* diaphragm, inspiratory pressure-generating capacity is remarkably preserved across ventilatory (chemoactivated breathing and augmented breaths) and non-ventilatory behaviours (sustained tracheal occlusion) [13], revealing a role for accessory and auxiliary muscles in support of peak inspiratory pressure generation in dystrophic disease [80]. We compared oesophageal pressure and respiratory muscle EMG (diaphragm and external intercostal) measurements in *mdx* mice following supplementation with 1% NAC to our previously published results. Values for oesophageal pressure during baseline (−9.3 ± 3.4 *versus* −9.2 ± 3.5 cmH_2_O; *mdx* + NAC *versus mdx*) and maximal inspiratory efforts (−55.4 ± 13.3 *versus* 59.7 ± 11.8 cmH_2_O; *mdx* + NAC *versus mdx*) were equivalent between *mdx* and *mdx* + NAC mice. Furthermore, NAC supplementation had no effect on diaphragm and external intercostal EMG activity in *mdx* mice during baseline conditions (diaphragm: 6 ± 4 *versus* 6 ± 5 A.U.; EIC: 3 ± 1 *versus* 3 ± 2 A.U.; *mdx* + NAC *versus mdx*) and peak inspiratory efforts during airway obstruction (diaphragm: 30 ± 17 *versus* 25 ± 15 A.U.; EIC: 18 ± 13 *versus* 14 ± 9 A.U.; *mdx* + NAC *versus mdx*). Decreased maximal respiratory EMG activities in *mdx* mice is likely reflective of neuromuscular junction impairment [81,82], which appears not to have been affected by NAC administration, but this requires further investigation.

Measures of somatic growth such as tibia length and mass were equivalent across all three groups. In terms of organ measures, *mdx* mice had heavier right and left heart ventricles and spleens than age-matched wild type mice. This is consistent with our previous observations [17,42]. Brown and white adipose tissues were decreased in *mdx* compared with wild type; NAC supplementation resulted in a modest increase in brown adipose tissue mass. Collectively, these are interesting findings in the context of previous research addressing potential side effects of NAC particularly in respect of reductions in body mass [27,75,83]. Left ventricle mass was unaffected by NAC supplementation in *mdx* mice, while right ventricle mass was decreased. This could potentially be a beneficial effect of NAC acting to reduce cardiac muscle hypertrophy or collagen deposition in the right ventricle. Overall, NAC supplementation had no major effects on body mass, indices of somatic growth, organ mass and brown and white adipose tissue mass, demonstrating no adverse effects of our relatively short intervention. Changes in right ventricle and spleen mass were observed and it will be important to discern in future work if these changes are adaptive or maladaptive outcomes in dystrophic tissue in response to NAC. Future work should widen the scope of assessment of the effects of NAC supplementation on physiological systems and incorporate assessments of NAC in other animal models of muscular dystrophy. It will be important to establish the efficacy of NAC supplementation in models of advanced dystrophic disease affecting ventilatory performance.

## 5. Conclusions

In summary, *mdx* mice have diaphragm muscle weakness and myofibre remodelling, immune cell infiltration and collagen deposition. Pro-inflammatory cytokines are increased in diaphragm muscle and plasma samples from *mdx* mice. Supplementation of *mdx* mice with NAC resulted in functional improvements of the diaphragm muscle, immune cell infiltrates and collagen deposits were reduced in the diaphragm, and systemic cytokine concentrations were reduced and diaphragm Nrf2 mRNA expression was increased. There were also sizeable reductions in the concentrations of IL-1β and IL-6 in *mdx* diaphragms following NAC supplementation. These findings are important and relevant to the development of adjunctive therapies with an ambition to alleviate muscle pathology in human dystrophinopathies.

## Figures and Tables

**Table 1:** Real-time ready catalog and custom assays used for cDNA amplification.

Gene Title	Gene Symbol	Assay ID
Atrogin1	Fbxo32	317844
BNIP3	Bnip3	311465
Catalase	Cat	310718
GABARAPL1	Gabarapl1	317923
GPX2	Gpx2	318630
HPRT	Hprt	307879
IGF1	Igf1	313359
LC3B	Map1lc3b	317920
MEF2C	Mef2c	318629
Myogenin	Myog	313501
Myostatin	Mstn	318626
MyoD	Myod1	313570
MuRF1	Trim63	317843
NFκB	Nfkb1	300085
NOX2	Cybb	317885
Nrf2	Nfe2l2	313377
PARK2	Park2	317264
PINK1	Pink1	331846
Sirtuin1	Sirt1	310480
SOD1	Sod1	310738
SOD2	Sod2	310295

**Table 2:** Body mass, tibia mass and length, organ mass and adipose tissue mass. Definition of abbreviations: NAC, N-acetylcysteine; RV, right heart ventricle; LV, left heart ventricle; BAT, brown adipose tissue; eWAT, epididymal white adipose tissue. *Mdx* + NAC group received 1% N-acetylcysteine in the drinking water for 14 days. Data are shown as mean ± SD and were statistically compared using one-way ANOVA with Newman-Keuls *post hoc* test, or Kruskal-Wallis with Dunn’s *post hoc* test. * denotes *mdx* different from corresponding wild type group; * *P* < 0.05, ** *P* < 0.01, *** *P* < 0.001 and **** *P* < 0.0001. ^$^ denotes *mdx* + NAC different from corresponding *mdx* group; ^$^ *P* < 0.05 and ^$$^ *P* < 0.01.

	Wild Type (*n* = 10)	*mdx* (*n* = 10)	*mdx* + NAC (*n* = 10)	*P* Value
Body mass (g)	24.8 ± 1.5	26.0 ± 0.8	26.7 ± 1.6	*P* = 0.0194
Tibia length (mm)	16.3 ± 0.7	16.4 ± 1.1	16.2 ± 0.7	*P* = 0.9528
Tibia mass (mg)	40.7 ± 4.1	39.9 ± 6.4	38.8 ± 4.5	*P* = 0.7019
RV (mg)	20.6 ± 3.6	28.9 ± 2.8 ****	25.6 ± 2.2 ^$^	*P* < 0.0001
LV (mg)	70.2 ± 5.1	82.7 ± 7.9 **	79.5 ± 7.8	*P* = 0.0048
RV:LV	0.29 ± 0.06	0.35 ± 0.05 *	0.33 ± 0.05	*P* = 0.0411
Spleen (mg)	58.4 ± 7.2	99.0 ± 10.1 ****	119.9 ± 25.0 ^$$^	*P* < 0.0001
Kidney (mg)	301.7 ± 26.4	322.9 ± 64.2	359.8 ± 31.0	*P* = 0.004
Lung (mg)	136.4 ± 14.4	150.9 ± 36.2	159.3 ± 36.2	*P* = 0.4124
BAT (mg)	64.2 ± 9.0	43.8 ± 14.2 **	53.5 ± 7.5	*P* = 0.0048
eWAT (mg)	407.8 ± 43.2	216.2 ± 57.3 ***	217.9 ± 58.3	*P* < 0.0001

**Table 3:** Baseline ventilation in conscious mice. Definition of abbreviations: *f*_R_, respiratory frequency; NAC, N-acetylcysteine; *V*_E_, minute ventilation; *V*_T_, tidal volume. *Mdx* + NAC group received 1% N-acetylcysteine in the drinking water for 14 days. Data are shown as mean ± SD and were statistically compared using one-way ANOVA with Newman-Keuls *post hoc* test, or Kruskal-Wallis with Dunn’s *post hoc* test. * denotes *mdx* different from corresponding wild type group; * *P* < 0.05.

	Wild Type (*n* = 10)	*mdx* (*n* = 8)	*mdx* + NAC (*n* = 10)	*P* Value
**Normoxia (21% O_2_)**				
*f*_R_ (brpm)	196 ± 15	211 ± 14	205 ± 16	*P* = 0.1136
*V*_T_ (mL g^−1^ body mass)	0.007 ± 0.001	0.006 ± 0.001	0.006 ± 0.001	*P* = 0.1295
*V*_E_ (mL g^−1^ min^−1^)	1.31 ± 0.15	1.28 ± 0.25	1.29 ± 0.10	*P* = 0.8891
Hypercapnic-Hypoxia (10% O_2_ & 6% CO_2_)				
*f*_R_ (brpm)	366 ± 28	360 ± 61	369 ± 15	*P* = 0.8817
*V*_T_ (mL g^−1^ body mass)	0.014 ± 0.002	0.011 ± 0.002 *	0.012 ± 0.001	*P* = 0.0147
*V*_E_ (mL g^−1^ min^−1^)	5.01 ± 0.98	3.99 ± 1.24	4.56 ± 0.47	*P* = 0.0903
Body mass (g)	24.8 ± 1.5	26.1 ± 0.8	26.7 ± 1.6	*P* = 0.0222

**Table 4:** Oesophageal pressure and diaphragm and T3 external intercostal (EIC) EMG activities during baseline and tracheal occlusion in anaesthetized mice. Definition of abbreviations: NAC, N-acetylcysteine; dia, diaphragm; EIC, external intercostal; NAC, N-acetylcysteine; A.U., arbitrary units. *Mdx* + NAC group received 1% N-acetylcysteine in the drinking water for 14 days. Sustained tracheal occlusion was reported as the average of five successive peak breaths. Data are shown as mean ± SD and were statistically compared using one-way ANOVA with Newman–Keuls *post hoc* test, or Kruskal-Wallis with Dunn’s *post hoc* test. * denotes *mdx* different from corresponding wild type group; ** *P* < 0.01.

	Wild Type (*n* = 10)	*mdx* (*n* = 10)	*mdx* + NAC (*n* = 10)	*P* Value
	**Baseline**			
Inspiratory pressure (cmH_2_O)	−9.0 ± 3.0	−9.2 ± 3.5	−9.3 ± 3.4	*P* = 0.9843
Dia EMG Amplitude (A.U.)	9.6 ± 6.7	5.5 ± 5.3	5.5 ± 3.7	*P* = 0.0907
T3 EIC EMG Amplitude (A.U.)	6.2 ± 2.7	3.3 ± 2.0 **	3.2 ± 1.1	*P* = 0.0027
	**Obstruction**			
Inspiratory pressure (cmH_2_O)	−67.5 ± 10.2	−59.7 ± 11.8	−55.4 ± 13.3	*P* = 0.0954
Dia EMG Amplitude (A.U.)	38.6 ± 16.2	25.0 ± 14.6	30.4 ± 16.8	*P* = 0.1741
T3 EIC EMG Amplitude (A.U.)	43.7 ± 19.9	14.3 ± 9.3 **	18.1 ± 12.5	*P* = 0.0011

**Table 5:** Respiratory measurements in anaesthetized mice in vivo. Definition of abbreviations: ETCO_2_, end-tidal carbon dioxide; *f*_R_, respiratory frequency; NAC, N-acetylcysteine; PIF, peak inspiratory flow; PEF, peak expiratory flow; SpO_2_, peripheral capillary oxygen saturation; *V*_E_, minute ventilation; *V*_T_, tidal volume. *Mdx* + NAC group received 1% N-acetylcysteine in the drinking water for 14 days. Data are shown as mean ± SD and were statistically compared using one-way ANOVA or Kruskal–Wallis.

	Wild Type (*n* = 10)	*mdx* (*n* = 7–8)	*mdx* + NAC (*n* = 8–10)	*P* Value
**Baseline Post-Vagotomy (60% O_2_)**				
*f*_R_ (brpm)	65 ± 21	61 ± 12	69 ± 19	*P* = 0.7421
*V*_T_ (mL g^−1^)	0.028 ± 0.005	0.029 ± 0.003	0.030 ± 0.008	*P* = 0.8468
*V*_E_ (mL g^−1^ min^−1^)	1.8 ± 0.4	1.8 ± 0.3	1.9 ± 0.2	*P* = 0.5651
PIF (mL g^−1^ sec^−1^)	1.10 ± 0.22	1.14 ± 0.12	1.12 ± 0.41	*P* = 0.9498
PEF (mL g^−1^ sec^−1^)	1.08 ± 0.17	1.17 ± 0.27	1.20 ± 0.26	*P* = 0.5177
ETCO_2_ (mmHg)	53.3 ± 3.4	49.6 ± 7.9	52.9 ± 3.3	*P* = 0.3033
SpO_2_ (%)	94.9 ± 2.3	95.1 ± 2.4	93.5 ± 4.1	*P* = 0.5320
Hypercapnic-Hypoxia (15% O_2_ & 6% CO_2_)				
*f*_R_ (brpm)	57 ± 17	52 ± 12	62 ± 16	*P* = 0.4772
*V*_T_ (mL g^−1^)	0.041 ± 0.007	0.046 ± 0.005	0.043 ± 0.009	*P* = 0.604
*V*_E_ (mL g^−1^ min^−1^)	2.3 ± 0.7	2.4 ± 0.6	2.6 ± 0.4	*P* = 0.6929
PIF (mL g^−1^ sec^−1^)	1.43 ± 0.23	1.45 ± 0.14	1.43 ± 0.35	*P* = 0.611
PEF (mL g^−1^ sec^−1^)	1.15 ± 0.19	1.38 ± 0.18	1.29 ± 0.20	*P* = 0.0655
SpO_2_ (%)	59.8 ± 5.9	64.6 ± 9.8	55.7 ± 6.3	*P* = 0.0865

**Table 6:** Ex vivo respiratory muscle contractile kinetics. Definition of abbreviations: ½ RT, half-relaxation time; CSA, cross-sectional area; CT, contraction time; L_o_, optimum length; NAC, N-acetylcysteine; P_t_, twitch force; P_o_, tetanic force; S_max_, maximum unloaded shortening; V_max_, maximum shortening velocity. *Mdx* + NAC group received 1% *N*-acetylcysteine in the drinking water for 14 days. Data are shown as mean ± SD and were statistically compared using one-way ANOVA with Newman-Keuls *post hoc* test, or Kruskal-Wallis with Dunn’s *post hoc* test. * denotes *mdx* different from corresponding WT group; ** *P* < 0.01 and *** *P* < 0.001. ^$^ denotes *mdx* + NAC different from corresponding *mdx* group; ^$^ *P* < 0.05 and ^$$^ *P* < 0.01.

	Wild Type (*n* = 10)	*mdx* (*n* = 8–9)	*mdx* + NAC (*n* = 10)	*P* Value
CT (ms)	17.2 ± 2.6	18.1 ± 4.0	19.2 ± 2.5	*P* = 0.3816
½ RT (ms)	19.9 ± 6.9	21.7 ± 9.0	22.5 ± 4.2	*P* = 0.7034
Smax (cm)	0.39 ± 0.08	0.23 ± 0.06 **	0.36 ± 0.05 ^$^	*P* = 0.0014
Smax (L L_o_^−1^)	0.37 ± 0.06	0.31 ± 0.08	0.35 ± 0.03	*P* = 0.1016
Vmax (cm s^−1^)	4.1 ± 1.0	2.3 ± 1.2	4.4 ± 1.0 ^$^	*P* = 0.0184
Vmax (Lo s^−1^)	4.0 ± 1.0	3.2 ± 1.7	4.2 ± 0.7	*P* = 0.3223
L_o_ (cm)	1.1 ± 0.1	0.8 ± 0.1 ***	1.0 ± 0.1 ^$$^	*P* = 0.0004
